# Implications of Sex Differences on the Treatment Effectiveness in Heart Failure with Reduced Ejection Fraction Related to Clinical Endpoints and Quality of Life

**DOI:** 10.1007/s11897-023-00638-6

**Published:** 2023-12-07

**Authors:** D. Aydin, Y. Allach, J. J. Brugts

**Affiliations:** Department of Cardiology, Erasmus University Medical Centre, 3015 Rotterdam, The Netherlands

**Keywords:** Heart failure, Men, Women, Sex, HFrEF, QOL, Treatment

## Abstract

**Purpose of the Review:**

This narrative review will emphasize the necessity for more female enrollment in heart failure (HF) trials and proposes future investigations regarding optimal dosages. Ultimately, a deeper understanding of the unique pathophysiology and medication responses in both men and women is crucial for effective HF management and may improve the quality of life in women.

**Recent Findings:**

An analysis of 740 cardiovascular studies reveals that women make up only 38.2% of participants on average. Regarding to trials testing the effectiveness of HF medications, women’s involvement are as low as 23.1%. While current guidelines lack sex-specific treatment recommendations, emerging research suggests differential medication dosages could be beneficial. Studies indicate that women may achieve comparable outcomes with lower doses of certain medications (angiotensin-receptor blockers) compared to men, signaling potential for more tailored dosing approaches.

**Summary:**

We advocate that the next step in HF research should prioritize the importance of tailoring treatment for HF patients by taking into account the variations in drug absorption and distribution among women.

## Introduction

Women are underrepresented in clinical trials, despite the fact that there is an equal ratio in the prevalence of heart failure (HF) [1]. An extensive review on female enrolment in 740 cardiovascular studies revealed that the participation of women in these studies was as low as 38.2% in general [2]. Additionally, a systematic review and meta-analysis by Danielson et al. that investigated sex variations in trials that tested the effectiveness of pharmacological therapy in HF even showed that only 23.1% of the study population consisted of women [3]. In the context of patients with HF and reduced ejection fraction (HFrEF), Schroeder and colleagues conducted a comparative analysis of clinical characteristics and outcomes between randomized clinical trials (RCTs) and HF registries, stratified by sex. Their findings indicate a significant inequality in HFrEF RCTs across sexes, with women exhibiting considerably lower rates of trial participation (22% compared to 78% in men) [4•]. In contrast, the real-world HF registry, CHECK HF, exhibit a female participation rate of 33.6% among patients with HFrEF [5].

Current research and recommendations from guidelines have been based largely on men. To date, there is no distinction between HF treatment for men and women in the current recommendations. However, recent research has demonstrated that for women, a lower dose of some HF medications, such as angiotensin-converting-enzyme (ACE) inhibitors, angiotensin-receptor blockers (ARBs), and beta (β-) blockers, can be sufficient to achieve the same prognostic effects concerning the composite endpoint of time to all-cause mortality or hospitalization as compared to men with full dose [6]. Interestingly, the study discovered that women had a 30% lower risk for the composite endpoint while using only 50% of the prescribed dosage of the aforementioned drugs than men did when using 100% of the recommended dosage. This raises the question whether unisex dosages are still appropriate. Till now, no clear follow-up on this study finding has been given.

Sex disparities in reaction to HF therapy have previously been described. According to several studies, women frequently report more severe side effects from their HF medication than men do [7–11]. Recent research findings indicate that there are notable sex-related variances in plasma volume between women and men, which can consequently impact medication distribution and concentration within the bloodstream. These differences have the potential to influence the effectiveness and potential toxicity of drugs. Additionally, variances in body composition, such as higher levels of subcutaneous fat in women and higher levels of visceral fat in men, may contribute to differences in drug distribution and metabolism as well. Furthermore, sex-related disparities in drug metabolism enzymes and renal clearance mechanisms can significantly influence the pharmacokinetics of HF medications. An illustrative example includes variations in the activity of cytochrome P450 enzymes involved in drug metabolism, which may differ between sexes and subsequently lead to differing rates of drug clearance [12]. This might imply that the present dose prescription in the HF guidelines does not adequately address the needs of the female sex and could explain why women generally benefit more from a lower dose of HF medication. There is recognition of the importance of tailored therapy, which has the potential to significantly improve the health of women in particular [13]. When tailored therapy is taken into consideration, this might aid in more precise prevention, management, and treatment of women with HF.

This narrative review aims to present a comprehensive summary of existing research demonstrating sex-based in treatment response among patients with HFrEF. Furthermore, we will delve into the pathophysiology of HF in both men and women, as well as the pharmacokinetics of recommended medications according to guidelines.

## Quality of Life in HF

The prognosis of HFrEF varies depending on the severity of the condition and the underlying cause of the disease. With appropriate treatment, including recommended guideline medications such as ACE inhibitors, β-blockers and diuretics, as well as lifestyle changes, many individuals with HFrEF are able to manage their symptoms and maintain a good QoL. However, studies suggest that women with HF tend to have a lower QoL compared to men with the same condition [14–17]. Interestingly, women report a lower score for the Kansas City Cardiomyopathy Questionnaire (KCCQ) than men [14•]. They do, however, show a similar improvement of QoL. This could be due to various factors such as the cause of HF, the way symptoms manifest, experience of side effects related to drugs, under-treatment, the availability and/or access to healthcare services, and social support. For example, the study by Heo et al. revealed that women suffering from HF scored lower on QoL measures than men, particularly in the areas of physical function, social function, and emotional well-being [17]. The study also showed that women tend to report more symptoms, such as fatigue and shortness of breath, which may contribute to their lower QoL.

## Selected Studies

**Fig. 1:**
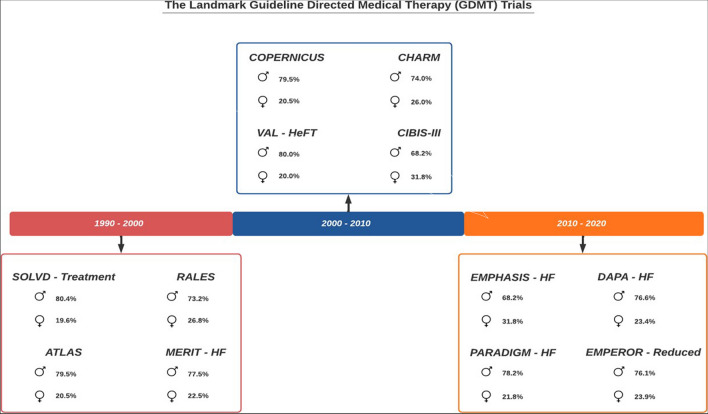
Overview of men and women participants included in the landmark guideline directed medical therapy(GDMT)HF trial

This review aims to elucidate the potential sex-related differences in treatment efficacy for the cornerstone medications used in the management of HF: β-blockers, renin-angiotensin-system inhibitors (RASi), mineralocorticoid receptor antagonists (MRA), and sodium glucose co-transporter 2 (SGLT2) inhibitors. We will highlight several studies that have investigated these differences. Furthermore, we will incorporate the findings from landmark trials published in the past 15 years pertaining to HF therapy, as outlined in Figure 1. Given that the majority of trials have employed a composite endpoint of cardiovascular or all-cause death along with HF hospitalizations, our focus will primarily be on these endpoints. Additionally, recognizing the considerable symptom burden and functional limitations experienced by HF patients, as well as their expressed value of improving QoL we have also included QoL as an endpoint for evaluation.

## Drug Treatment and Clinical Endpoints in HF According to Sex

### β-blockers

#### Pharmacokinetics of β-blockers

One of the major therapy strategies for treating patients with chronic HF, and particularly those with HFrEF, is the use of β–blockers [18]. In order to exert their effects, β–blockers bind to the β-adrenoceptors found in tissues such as cardiac nodal tissue and prevent the circulating hormones norepinephrine and epinephrine from attaching to these receptors. More specifically, the β-adrenoceptors are connected to proteins known as Gs-proteins, which activate adenylyl cyclase and cause it to convert adenosine triphosphate (ATP) into cyclic adenosine monophosphate (cAMP). Through this, calcium eventually enters the cell at a higher rate, promoting the release of calcium by the sarcoplasmic reticulum of the heart, increasing contractility [19, 20]. The pharmacokinetics of HF medications have been studied in literature with regard to sex variations. Regarding β–blockers, these variances have also been shown [6, 21, 22]. Previous research has demonstrated that hormones such as estrogens and progesterone have the capability to inhibit the cardiac expression of β1-adrenoceptors, thereby reducing β-adrenergic-mediated stimulation. This exerts cardio protective effects and may provide an explanation for the observed variations in the pharmacodynamics of β–blockers with respect to sex [23]. Women typically have distinct side effects than men when using β–blockers, which may have a number of clinical causes, including greater nerve activity, higher cardiac noradrenaline spillover, and increased β2-adrenoreceptor sensitivity [24–26]. Additionally, there are pharmacokinetic variations between the sexes that might account for the disparities in how the bodies of both sexes react to β–blockers [23, 27, 28]. For example, because of their larger Cmax and AUC as well as what appears to be a poorer oral clearance, women generally experience a higher drug exposure than men [29, 30]. There has also been substantial evidence to support the notion that men exhibit elevated activity of the CYP2D6 enzyme, which plays an important role in the metabolism of β–blockers. The increased expression of the CYP2D6 enzyme in men, attributed to elevated testosterone levels, has been proposed as the underlying factor contributing to enhanced drug clearance [22, 29, 31].

#### Sex Differences in the Use of β–blockers

Although β-blockers are an essential medication in HF therapy, as mentioned earlier, there is currently no differentiation in dosage between men and women. However, this is surprising since pharmacokinetic differences can cause women to experience greater systemic exposure than men when using the same β-blocker dosage. To achieve the same systemic dosage, measured as the area under the concentration-time curve (AUC), women may require only half the dosage of the β-blocker metoprolol used in men [30]. Danielson et al. conducted a meta-analysis to examine whether there are differences in the effect of guideline-recommended therapy for HFrEF between men and women [3]. The study showed that treatment with β-blockers reduced mortality risk in both sexes, with a hazard ratio (HR) of 0.71 (CI 0.59–0.86) in men and 0.87 (CI 0.73–1.03) in women. However, no significant differences were found when comparing the two sexes, with an overall HR of 0.72 (0.60–0.85, *P* interaction = 0.345), which was also the case for the combined outcome of mortality and/or hospitalization for HF (HR 0.76 (0.64–0.90, *P* interaction = 0.650)). Kotecha et al. conducted another meta-analysis, which showed similar findings to Danielson et al.'s study. This meta-analysis, which included 11 trials, confirmed that there is no difference in the efficacy of β-blockers between men and women with HFrEF [32]. This finding was contrary to Santema et al.’s observational cohort study which found that women with HFrEF using half of the guideline-recommended doses of β-blockers and ACE or ARB had a lower risk of adverse outcomes compared to men. The researchers suggest that a more tailored approach to HF medication should be considered for women since they may require lower dosages [6]. Overall, the trials investigating β-blockers did not consistently demonstrate significant heterogeneity of treatment effects on clinical endpoints between men and women; however, some suggest lower dose for women.

### Renin-Angiotensin-System Inhibitors (RASi)

#### ACE Inhibitors

##### Pharmacokinetics of ACE Inhibitors

In patients with HFrEF, the use of ACE inhibitors is a common and essential component of HF treatment. Angiotensin II, the primary effector molecule of the renin-angiotensin-aldosteronsystem (RAAS), is suppressed by ACE inhibitors from being produced. This inhibiting impact raises sodium and urine excretion while decreasing cardiac output and eventually stimulating blood vessel dilatation [33]. Significant differences in the pharmacokinetics between the sexes have been described in literature [34]. The authors of the study of Zapater et al. found that women at the follicular phase showed lower minimum ACE activity following enalapril administration. The menstrual cycle, menopausem, and oral contraceptives for instance may affect the absorption and distribution of medications including ACE inhibitors. So, it should be noted that the aforementioned finding may be altered by the hormones produced by women [6]. The RAAS pathways show unique regulatory patterns influenced by gonadal hormones. In particular, androgens are known to upregulate these pathways, thereby increasing vasopressor activity, while estrogens have the opposite effect, reducing renin-angiotensin activity. A recent study conducted by Rogers et al. provides evidence of a distinct gender-specific response to ACE inhibitor and ARB therapies in relation to arterial stiffness. Notably, postmenopausal women experienced significant improvement in arterial stiffness with ACE inhibitor treatment, whereas ARB therapy did not yield such improvements. Conversely, among men, neither medication resulted in significant changes in arterial stiffness. These findings may be partially explained by the fact that the postmenopausal period is characterized by increased AT-1 receptor expression and elevated angiotensin II levels due to declining estrogen levels. As a result, these hormonal changes may reduce the effectiveness of specifically ARBs in postmenopausal women [35].

##### Sex Differences in the Use of ACE Inhibitors

In addition to variances in pharmacokinetics between men and women, disparities in side effects also exist. Several studies have reported adverse drug reactions (ADR) associated with ACE inhibitors, yet only five studies have specifically addressed sex-specific ADRs. Among these, three studies suggested a higher risk of ADRs in women, while two studies found no discernible difference [36]. To shed light on this hypothesis, a cross-sectional study conducted by Bots et al. investigated the reporting of ADRs associated with ACE inhibitors. The study revealed a disparity between the sexes, with women experiencing a greater number of ADRs. Specifically, women exhibited a 1.31-fold higher rate of ADRs (95% CI, 1.27–1.35) [37•]. Subsequently, two post hoc analyses conducted by Konstam et al. on the SOLVD trial, which examined the efficacy of enalapril in patients with HF and left ventricular dysfunction, further confirmed these findings. The researchers discovered that women in the enalapril group reported the side effect of coughing more frequently than men (10% vs. 4.2%; OR, 2.38) [38, 39]. These studies raise pertinent questions about whether women are receiving optimal treatment and whether a potential reduction in dose could be appropriate to mitigate the ADRs experienced by women.

Multiple studies have examined the impact of ACE inhibitors on clinical endpoints, including hospitalization for HF and all-cause mortality. In a meta-analysis conducted by Danielson et al., a risk reduction for mortality was observed in both men (HR 0.86, 95% CI 0.75–0.99) and women (HR 0.97, 95% CI 0.77–1.23). Moreover, no significant difference in interaction between the two sexes was found (HR 0.86, 95% CI 0.73–1.01) [3]. Another study by Golyar demonstrated that ACE inhibitors enhance survival rates in both men and women with chronic HF. However, the protective effects appear to be more pronounced in men. Notably, the decrease in mortality among men was more prominent compared to women, with a reduction of 29% versus 20%, respectively [40]. These findings align with previous studies that have provided evidence of a greater reduction in mortality among men compared to women [41, 42]. The study performed by Santema et al. however indicated that women with HFrEF who utilized only half of the recommended doses of ACE inhibitors had a reduced chance of experiencing adverse outcomes compared to men [6]. Overall, these studies indicate that ACE inhibitors exhibit beneficial effects on survival in individuals with HF, but the magnitude of the effect may vary between men and women, with a potentially stronger impact observed in men in the majority of the studies.

#### ARBs

##### Pharmacokinetics of ARBs

Angiotensin-receptor blockers (ARBs), also known as angiotensin II receptor antagonists, constitute a pharmaceutical class used in the management of HF that acts by modulating the renin-angiotensin-aldosterone system (RAAS). These agents exert their therapeutic effects by inhibiting the actions of angiotensin 2, a hormone which influences various physiological processes such as aldosterone production and vasoconstriction. The effects of angiotensin 2 can lead to elevated blood pressure, ultimately causing detrimental consequences for both the cardiac and renal systems. ARBs function by obstructing the binding of angiotensin 2 to its specific receptor, namely the angiotensin II receptor type 1 (AT1), thereby diminishing the effects of angiotensin 2 [43]. It is noteworthy that ARBs differ from ACE inhibitors in their approach to reducing the actions of angiotensin. Specifically, ARBs operate by selectively blocking the angiotensin receptors, as opposed to inhibiting the enzymatic activity of angiotensin converting enzyme within the body. However, like previously described in “Pharmacokinetics of ACE Inhibitors,” the RAAS system does show differences in the way sex hormones act on this system.

##### Sex Differences in the Use of ARBs

Studies investigating the disparity in effectiveness between men and women regarding ARBs remain scarce. The sex differences between ARBs were assessed within the HEAAL trial comparing a low versus high dose of losartan in 1846 patients with symptomatic HF. The primary endpoint of the HEAAL trial was the composite of all-cause death or admission for HF. For this study regarding the sex differences the endpoints cardiovascular death, all cause death, the composite endpoint cardiovascular death or admission for HF and lastly the composite endpoint of all-cause death or all-cause hospitalization were analyzed. Ferreira et al. showed that men seem to benefit more from a high dose compared to a low dose, whereas in women there was no difference. They state that this effect may not be only driven by sex but also be caused by lower tolerability of high doses due to other factors, such as older age, poorer renal function, and more severe HF symptoms [44•]. In addition, as already stated, Santema et al. found that women reached the lowest risk for the composite endpoint all-cause mortality or HF hospitalization using only 40% of the dosage, whereas in men this was 100%. The study showed that for women a higher dose did not correlate with a decrease of the risk for the composite endpoint. These findings were validated using an independent HF cohort (ASIAN-HF). These remarkable results show that there exist differences between men and women regarding the dosage of ARBs, implicating that women may benefit from lower doses.

#### Angiotensin Receptor Neprilysin Inhibitors (ARNi)

##### Pharmacokinetics of ARNi

ARNi represents a pharmacological intervention employed in the management of HF by combining a neprilysin (NEP) inhibitor with an ARB. Natriuretic peptides (NPs) play an essential role in mediating favorable cardio-renal effects, such as vasodilation, inhibition of the RAAS system, suppression of the sympathetic nervous system, and aldosterone secretion. Increasing the biological activity of NPs, mainly when coupled with RAAS blockade, can have therapeutic advantages. Angiotensin receptor-neprilysin inhibition represents an innovative therapeutic approach based on increasing the availability of biologically active NPs by inhibiting their degradation through NEP inhibition, while simultaneously counteracting the harmful effects of persistent RAAS activation. Recent studies have demonstrated that the combined action of angiotensin receptor blockade and NEP inhibition holds promise in enhancing treatment of HF, and outperforms the efficacy of ACE inhibitors, which are commonly prescribed for the treatment of HFrEF [45–47]. Compared to men, women typically have lower body weight, a higher proportion of body fat, reduced liver blood flow, a decreased rate of glomerular filtration and a lower plasma volume [23, 48]. It's important to note that women may experience higher peak plasma concentrations when taking hydrophilic medications, like angiotensin receptor neprilysin inhibitors (ARNis) [12]. Which could explain why women tend to experience more adverse drug reactions from the medication than men do.

##### Sex differences in the Use of ARNi

Regarding the sex difference between ARNIs, the knowledge remains scarce. A small prospective study performed in ten different centers in Spain analyzed 427 HF patients using an ARNI. Vicent et al. analyzed the potential differences between men and women regarding the effect of sacubitril/valsartan. They show similar percentages of female participants as other HF studies, in which only a minority of their patients were women (29.5%). Furthermore, they did show that women had a significant functional class improvement (*P* = 0.05). The adverse effect due to sasubitril/valsartan, however, were not different between both sexes (*P* = 0.72) [49]. In a more extensive study (a meta-analysis), investigating this study and four other cohort studies, Nuechterlein et al. investigated major adverse outcome such as all-cause death, cardiovascular death, HF hospitalization, hyperkalemia, and hypotension. The meta-analysis showed that the here above mentioned adverse events were similar in men and women. They do however state more research is needed to do more future research including more women and with a longer follow up duration [50]. Overall, the current studies do not show significant differences between both sexes regarding adverse events.

### Mineralocorticoid Receptor Antagonist (MRA)

#### Pharmacokinetics of MRA

MRA is a component of the current HF therapy guidelines as a standard of care in the management of HF. This diuretic medication primarily affects the kidney by blocking the action of the hormone aldosterone at the mineralocorticoid receptor. As a result, salt reabsorption is prevented, resulting in water excretion. This effect is beneficial in reducing the symptoms of HF, particularly volume overload [51]. However, without a clear rationale, women with HFrEF receive MRA less frequently and at lower doses than men [52, 53]. Moreover, the paucity of evidence on the sex differences in the efficacy and safety of HF therapeutic interventions leads to poor appropriateness.

#### Sex Differences in the Use of MRA

For the effect of MRA’s different conclusions were withdrawn in comparison to the other cornerstone HF medications. In a pooled analysis by Rossello et al. where three MRA trials were compared it was shown that the characteristics of women such as age, body mass index, NYHA class and glomerular filtration rate did differ a lot from men. However the primary outcome of cardiovascular death or HF hospitalization did not show a significant difference between men and women (HR 0.70 (95% CI 0.64–0.76). Men showed similar beneficial effects of MRA (HR 0.69; 95% CI 0.62–0.77) as women (HR 0.71; 95% CI 0.60–0.83) [54]. Also, after adjusting for potential confounders the treatment benefit of MRA did not change considerably. Another meta-analysis, performed by Wang et al. reported that in women with HFrEF the efficacy of guideline-directed medical therapy (GDMT) did not show a significant benefit (RR 0.77, 95% CI 0.52–1.16). These findings were comparable in men MRA (0.79, 95% CI 0.69, 0.91). Similar effect size but different uncertainty around the estimate, point out an underrepresentation of women in these studies. This may be due to the low number of MRA studies or the lower percentage of MRA usage in men and women. These findings should therefore be interpreted with some caution and simultaneously advocate for further investigation. In conclusion, the current body of evidence examining the different treatment effects of MRA in men and women has yielded inconsistent results.

### Sodium Glucose Co-Transporter 2 (SGLT2) Inhibitors

#### Pharmacokinetics of SGLT2

Another drug that has a cardiovascular beneficial outcome in patients with HF is the SGLT2 inhibitors [55]. SGLT2 inhibitors were originally designed to treat hyperglycemia in patients with type 2 diabetes. However, SGLT2 inhibitors have been taken in the current HF therapy guidelines showing that their effects lead to a reduced risk for HF hospitalization. SGLT2 inhibitors function through a novel mechanism of reducing renal tubular glucose reabsorption, producing a reduction in blood glucose without stimulating insulin release. In HF they act by several processes such as reducing interstitial fluid, blood pressure reduction and increasing circulating ketone levels [56]. SLGT-2 is a very effective treatment for HF regardless of sex. When compared to men, women tend to experience less benefit from these drugs [57•]. This can be attributed to the fact that as women enter the menopausal stage, the protective effects diminish due to a significant decrease in estrogen levels associated with reproductive aging. Moreover, it is important to consider the impact of sex on the way SGLT-2 inhibitors work in the body. As mentioned earlier, women typically have lower plasma volume and a higher body fat percentage compared to men. Notably, this higher body fat content can lead to a higher volume of distribution of lipophilic drugs like SGLT-2 inhibitors [58].

#### Sex Differences in the Use of SGLT2i

Although initially prescribed primarily as a diabetic medication, SGLT inhibitors have demonstrated their efficacy in patients with HF in recent years. Previous studies have indicated that among individuals with diabetes mellitus, the reduction in major adverse cardiac events (MACE) achieved with SGLT2 inhibitors was comparatively less pronounced in women than in men (HR 0.88, 95% CI 0.77–1.00, *P* = 0.06, vs. HR 0.90, 95% CI 0.83–0.97, *P* = 0.006, respectively). However, the available research on the favorable impact of SGLT2 inhibitors on cardiovascular events in the HF population remains limited. Furthermore, SGLT2i are prescribed in a fixed dose, unlike the other HF medications that are up titrated. Nonetheless, a recently conducted meta-analysis by Rivera et al., investigating the effect of SGLT2 inhibitors on cardiovascular outcomes (specifically cardiovascular death and hospitalization for HF), concluded that SGLT2 inhibitors reduce the risk of these events in patients with HF irrespective of gender. However, the magnitude of the drug's benefits was observed to be comparatively less prominent in women (OR 1.32, 95% CI: 1.17–1.48) [57•]. The researchers indicate that this may be related to different reasons such as pharmacokinetics, clinical characteristics and representation of women in the trials.

Furthermore, in the meta-analysis conducted by Zannad et al., two studies investigating the effects of empagliflozin and dapagliflozin in patients with HFrEF were included, specifically examining the time to all-cause death and the combined risk of cardiovascular death or hospitalization for HF. This meta-analysis revealed a risk reduction in both men (HR 0.76 (0.68–0.85)) and women (HR 0.68 (0.56–0.84)), with a *P* value for interaction of 0.37 [59]. Nevertheless, it is important to interpret these findings cautiously, as the authors did not account for multiple testing in their analysis.

## Discussion

According to the latest European Society of Cardiology (ESC) guidelines for the diagnosis and treatment of acute and chronic HF, no sex-specific treatment recommendations exits on GDMT. While intuitively in clinical practice we do recognize the lower overall effect of GDMT, higher number of intolerance and side-effects in women at high dosages, we have not found any clear signal on an existing heterogeneity in treatment efficacy of GDMT classes BB, RASi, MRA, and SGLT2 in contemporary HF trials. Women may exhibit distinct pharmacokinetic patterns related to the absorption of cornerstone HF medications, potentially influencing their sensitivity to treatment effects. An overview of the sex differences regarding pharmacokinetics in HF cornerstone therapy can be found in Table 1. For instance, within the context of HF medication metabolism, it has been observed that women tend to have increased activity of cytochrome P450 (CYP) 3A4 and 2D6 enzymes, of which both play an important role in the metabolism of several HF drugs. Conversely, women often display reduced activity of the transporter protein P-glycoprotein. Consequently, the higher hepatic clearance observed for substrates of CYP3A4 in women could possibly arise from the reduced activity of P-glycoprotein [12, 60–63]. Furthermore, women tend to have a lower renal blood flow, estimated glomerular filtration rate (eGFR), and a reduced capacity for tubular secretion and/or reabsorption in comparison to men. As a consequence, drugs primarily eliminated from the body in an unchanged state through urinary excretion tend to clear at a slower pace in women. As a result, when the same doses of these drugs are given to both women and men, women can end up with higher drug levels in their system. This discrepancy in drug clearance leads to a higher likelihood of experiencing adverse drug reactions among women. Given this situation, it becomes crucial to consider different dosing approaches for women and men who have HF. Specifically, for HF drugs that are recommended in guidelines and are excreted mainly through the kidneys, like ACE inhibitors, bisoprolol, and diuretics [21, 61, 62]. However, the current guidelines do not address these differences and do not provide specific dosage recommendations for women or men.

**Table 1:** pharmacokinetic sex differences in heart failure drug therapy

Drugs class	Sex differences in pharmacokinetics	References
β- blockers	Lower oral clearance in women	[29, 30]
	Larger Cmax and AUC in women	
	Greater nerve activity, higher cardiac noradrenaline spillover and increased β2-adrenoreceptor sensitivity in women	[24–26]
	Elevated activity of the CYP2D6 enzyme in men	[22, 29, 31]
ACE-inhibitors	No significant pharmacokinetic sex differences RAAS pathway can be influenced by gonadal hormones in both sexes (e.g. androgens upregulate these pathways, where estrogens reduce them)	[6, 34]
ARB’s	No significant pharmacokinetic sex differences RAAS pathway can be influenced by sex hormones	[6, 34]
ARNI	More adverse drug reactions in women due to: lower body weight, a higher proportion of body fat, reduced liver blood flow, a decreased rate of glomerular filtration and a lower plasma volume	[23, 48]
	Higher peak plasma in women with hydrophilic drugs	[12]
MRA	No significant pharmacokinetic differences	
SGLT2-I	Lower plasma volume and higher body fat % in women	[58]
	Higher volume of distribution of lipophilic drugs	[58]

β- blocker: beta-blocker, ACE: angiotensin-converting-enzyme, ARB:Angiotensin-receptor blocker, ARNI: Angiotensin Receptor Neprilysin inhibitor, MRA: Mineralocorticoid receptor antagonist, SGLT2-i: Sodium Glucose Co-Transporter 2 inhibitors, RAAS: renin-angiotensin-aldosteronsystem, AUC: area under the curve

## Conclusion

The drugs examined in this review have demonstrated divergent efficacy profiles between men and women, as evidenced by their impact on various endpoints such as hospitalization for HF and all-cause mortality. The potential impact of the low representation of female participants in HF trials remains uncertain unless effective measures are implemented to enhance female enrollment in such trials. To gain clarity on this matter, it is essential to explore solutions that increase female participation levels and conduct more extensive investigations on female participants in GDMT drug classes, incorporating longer follow-up periods and possibly examining at varying (optimal) dose. A deeper understanding of the dynamic pathophysiological mechanisms involved in HF and the pharmacokinetic properties of HF medications is needed in men and women.
